# Association of small-fiber polyneuropathy with three previously unassociated rare missense SCN9A variants

**DOI:** 10.1080/24740527.2020.1712652

**Published:** 2020-02-05

**Authors:** Mary A. Kelley, Anne Louise Oaklander

**Affiliations:** a Department of Neurology, Massachusetts General Hospital, Harvard Medical School, Boston, Massachusetts, USA; b Department of Neurology, Dell Medical School at the University of Texas, Austin, Texas, USA; c Department of Pathology (Neuropathology), Massachusetts General Hospital, Harvard Medical School, Boston, Massachusetts, USA

**Keywords:** small fiber neuropathy, *SCN9A*, peripheral neuropathy, genetic neuropathy

## Abstract

**Background**: Small fiber polyneuropathy (SFN) involves ectopic firing and degeneration of small-diameter, somatic/autonomic peripheral axons. Causes include diabetes, inflammation and rare pathogenic mutations, including in *SCN9-11* genes that encode small fiber sodium channels.

**Aims**: The aim of this study is to associate a new phenotype—immunotherapy-responsive SFN—with rare amino acid–substituting *SCN9A* variants and present potential explanations.

**Methods**: A retrospective chart review of two Caucasians with skin biopsy confirmed SFN and rare *SCN9A* single nucleotide polymorphisms not previously reported in neuropathy.

**Results**: A 47-year-old with 4 years of disabling widespread neuropathic pain and exertional intolerance had nerve- and skin biopsy–confirmed SFN, with blood tests revealing only high-titer antinuclear antibodies and low complement C4 consistent with B cell dysimmunity. Six years of intravenous immunoglobulin (IVIg) therapy markedly improved sensory and autonomic symptoms and normalized his neurite density. After whole exome sequencing revealed a potentially pathogenic *SCN9A*-A3734G variant, sodium channel blockers were tried. Herpes zoster left a 32-year-old with disabling exertional intolerance (“chronic fatigue syndrome”), postural syncope and tachycardia, arm and leg paresthesias, reduced sweating, and distal hairloss. Screening revealed antinuclear and potassium channel autoantibodies, so prednisone and then IVIg were prescribed with great benefit. During 4 years of immunotherapy, his symptoms and function improved, and all abnormal biomarkers (autonomic testing and skin biopsies) normalized. Whole exome sequencing then revealed two nearby compound heterozygous *SCN9A* variants that were computer-predicted to be deleterious.

**Conclusions**: These cases newly associate three novel amino acid–substituting *SCN9A* variants with immunotherapy-responsive neuropathy. Only larger studies can determine whether these are contributory or coincidental, but they associate new variants with moderate or high likelihood of pathogenicity with a new highly related phenotype.

## RÉSUMÉ

**Contexte**: La polyneuropathie des petites fibres implique le déclenchement d’activité ectopique et la dégénérescence des axones périphériques somatiques et autonomes de petit diamètre. Ses causes comprennent le diabète, l'inflammation et de rares mutations pathogènes, notamment dans les gènes SCN9-11 qui codent les canaux sodiques des petites fibres.

**Objectifs**: Associer un nouveau phénotype de polyneuropathie des petites fibres répondant à l’immunothérapie à des variantes rares de SCN9A substituant des acides aminés et présenter des explications possibles.

**Méthodes**: Examen rétrospectif des dossiers de deux personnes de race blanche ayant subi une biopsie cutanée et présentant des polymorphismes mononucléotidiques de SCN9A rares qui n'avaient pas été signalés auparavant dans le cadre d'une neuropathie.

**Résultats**: Une homme de 47 ans souffrant depuis quatre ans de douleurs neuropathiques généralisées invalidantes et d'intolérance à l'effort a subi une biopsie des nerfs et de la peau qui a confirmé la polyneuropathie des petits fibres, les analyses sanguines ne révélant que des anticorps antinucléaires de haut niveau et un faible complément C4 correspondant à la dysimmunité des lymphocytes B. Six ans de traitement par immunoglobulines intraveineuses ont permis d'améliorer sensiblement les symptômes sensoriels et autonomes et de normaliser la densité de ses neurites. Après que le séquençage de l'exome entier ait révélé une variante de SCN9A-A3734G potentiellement pathogène, des inhibiteurs des canaux sodiques ont été essayee. Le zona a entrainé chez un homme de 32 ans une intolérance à l'effort invalidante (« syndrome de fatigue chronique »), une syncope posturale et une tachycardie, une paresthésie des bras et des jambes, une réduction de la transpiration et une perte de cheveux aux jambes. Le dépistage a révélé la présence d'auto-anticorps antinucléaires et de canaux potassiques, de sorte que la prednisone puis des immunoglobulines intraveineuses ont été prescrites, avec d’excellents résultats. Pendant quatre ans d'immunothérapie, ses symptômes et son fonctionnement se sont améliorés et tous les biomarqueurs anormaux (tests autonomes et biopsies cutanées) se sont normalisés. Le séquençage de l'exome entier a ensuite révélé deux variantes hétérozygotes de SCN9A composées à proximité et prédites par ordinateur comme étant délétères.

**Conclusions**: Ces cas associent trois nouvelles variantes de SCN9A substituant des acides aminés à une neuropathie répondant à l'immunothérapie. Seules des études de plus grande envergure peuvent déterminer s'il s'agit de facteurs contributifs ou de coïncidences, mais ces cas associent de nouvelles variantes ayant une probabilité modérée ou élevée de pathogénicité à un nouveau phénotype étroitement apparenté.

## Introduction

The term “polyneuropathy,” often shortened to “neuropathy,” refers to widespread damage to the body’s peripheral nerves. Small fiber neuropathy (SFN) refers to those neuropathies that predominantly or exclusively affect the thinnest fibers (axons): the unmyelinated C fibers, sympathetic and parasympathetic autonomic axons, and thinly myelinated A-δ sensory axons.^1^ In the past, somatic and autonomic neuropathies were classified separately, but when it was discovered that C fibers also have important effector and trophic functions—for example, controlling sweating, bone growth, immunocytes, and the microcirculation^2,3^—the aggregate “small fiber” terminology became preferred.

Because different types of small fibers express different arrays of neuropeptides and innervate many different types of cells, tissues, and organs, SFN typically presents with multiple symptoms. Its sensory symptoms include widespread neuropathic itch as well as chronic pain.^4^ Many organs can be affected by internal and autonomic SFN. The most common complaints are new difficulty performing usual activities (exertional intolerance and chronic fatigue), postural orthostatic hypotension and tachycardia (POTS),^5^ postprandial bloating or nausea, diarrhea and/or constipation (irritable bowel),^6^ urological complaints, and chronic daily headache.^7^ Patients with SFN often become disabled. If they receive inadequate validation, guidance, or treatment, it can leave them confused, frightened, and often depressed. SFN can have devastating psychological, economic, and quality-of-life consequences. At least some of the consequences of SFN can be mitigated by informed, compassionate caregivers. Recent reviews and the expected 2020 publication of the first global consensus case definition of SFN are increasing its visibility.^1,8^ This case definition and diagnostic criteria for “definite,” “probable,” and “possible” cases, although intended for research use, will inform medical practice as well.

Given the multiplicity and nonspecificity of small fiber symptoms, the new case definition includes a requirement for objective “biomarker” confirmation, at least for research studies. Small fibers are beyond the resolution of the diagnostic tests for large fiber neuropathy (nerve conduction and electromyographic study), so it will endorse skin biopsy from the standard lower leg location for confirming suspected SFN diagnoses. These skin biopsies can be performed in any medical setting and mailed in fixative to a nationally accredited neuropathology lab for quantification of epidermal neurite density (END) and interpretation of the normality of each patient’s biopsy. This requires statistical comparison of the measured END with the predicted normal END distribution that has been derived from hundreds of identically processed biopsies from healthy volunteers. These biopsies are the universally recommended objective test for SFN, with sensory nerve biopsy, quantitative autonomic function testing, and corneal confocal imaging as secondary choices.^9,10^ Biopsies can be removed in any health care setting, placed in a vial of fixative, and mailed to specialized neuropathology laboratories. Here they are vertically sectioned and immunolabeled against PGP9.5, the best pan-axonal marker.^11^ PGP9.5 darkens and magnifies the small fibers as they course through the tissues to permit END to be measured with endorsed methods.^9,10,12,13^ Skin biopsies can be repeated to track natural history or treatment outcomes. Although the slowest to conduct action potentials, small fibers have the highest regenerative capacity, so prompt diagnosis and initiation of disease-modifying treatment of SFN can permit substantial regeneration and recovery, particularly in the young and otherwise healthy, as is the case here.

Diabetes is the most common cause of SFN in developed countries, with cancer chemotherapies and other toxic exposures also well recognized.^1^ However, such common causes of neuropathy are usually quickly diagnosed by history, exam, or basic testing and then managed by ameliorating the cause when possible. Yet, 20% to 50% of patients with objectively confirmed SFN have no recognized medical risks; this is known as “cryptogenic” or “initially idiopathic” SFN. Many of these patients remain undiagnosed and untreated for years and in some instances for decades or throughout their entire lives.^14–18^ Evidence-based recommendations have been developed for blood test screening of patients with initially idiopathic small fiber neuropathy (iiSFN); a list is available at https://neuropathycommons.org/.^19^ Such screening identifies potential medical causes or contributors in nearly half of patients with iiSFN, and when a cause is identified it not only guides medical management but offers a welcome path forward to patients.^17,19,20^

The largest screening studies report elevated prevalence of serological markers of inflammation and dysimmunity in iiSFN,^19,20^ suggesting that some cases are associated with unrecognized inflammation directed at small fiber epitopes. Primary Sjögren’s syndrome (often seronegative^21^) is the most common systemic autoimmune disease associated with SFN, and efficacy of treatment with pooled intravenous immunoglobulins was recently reported.^22^ Other iiSFN appears to develop as part of ill-defined systemic dysimmune/inflammatory conditions, with selective immunoglobulin deficiencies particularly common,^1^ and reports have associated autoantibodies with dysautonomic SFN symptoms including POTS.^23,24^ In a small passive transfer study that injected sera from patients with “idiopathic” painful small fiber polyneuropathy to reproduce symptoms, physiology and pathology in mice provided direct support of the hypothesis that some cases of iiSFN are B cell mediated.^25^ However, the largest pool of evidence comes from the many cases and case series reporting efficacy of two immunotherapies effective for large fiber immune neuropathies: corticosteroids and/or pooled intravenous immunoglobulin (IVIg) for iiSFN.^22,26–28^

Pathogenic single nucleotide polymorphisms (SNPs) that alter proteins (nonconservative or missense mutations) preferentially expressed by small fibers are another increasingly discussed contributor to SFN. These include several of the historically named hereditary sensory and autonomic neuropathies (types 1 to 5), X-linked Fabry disease, autosomal-dominant transthyretin amyloidosis, and mutations affecting small fiber ion channels including the Na_v_1.6–1.9 sodium channels as well as the HCN2, TRPA1, TRPV4, and Piezo2 channels (reviewed in Oaklander and Nolano^1^). Ion channels are intermembrane gated pores that regulate voltage potential across the axolemma by controlling ion flow between the intracellular and extracellular compartments. Their openings and closings produce electrical signals that cause adjacent voltage-sensitive channels to open, thus initiating and propagating the action potentials that neurons use to transmit information. Of approximately 215 ion channels found in humans, 85 are linked to pain.^29^ The most important channels preferentially expressed on pain-sensing small fiber axons are the voltage-gated sodium (Na_v_) channel family: Na_v_1.7, Na_v_1.8, and Na_v_ 1.9. These are encoded for by the *SCN9A, 10A*, and *11A* genes, respectively.^30,31^ Various deterministic (Mendelian) autosomal-dominant gain-of-function and loss-of-function Na_v_ mutations have been associated with familial and sporadic pain and itch abnormalities, with *SCN9A* most commonly reported.^32–36^ There may be regional differences; a U.S. study of mixed neuropathy patients reported no correlations between the presence of Na_v_ variants and pain status,^37^ whereas a Dutch study of 1139 patients with pure clinically defined SFN reported that 11.6% harbored 73 potentially pathogenic variants in voltage-gated sodium channels, specifically 5.1% for *SCN9A*, 3.7% for *SCN10A*, and 2.9% for *SCN11A*.^38^ Among Dutch patients with neuropathy symptoms, only the presence of “erythromelalgia” and “warmth-induced pain” symptoms was more common in patients harboring Na_v_ variants.^38^

The potential for interactions between immune and genetic risks for SFN is largely unexplored, but these considerations are beginning in other conditions. Immune mechanisms are increasingly recognized as the proximate cause of neurological degeneration even in the presence of other insults, including trauma and Alzheimer’s disease.^39^ This article is one of the first discussion of potential genetic–immune interactions in patients with SFN.

## Case 1

A 47-year-old Caucasian laborer presented with 4 years of SFN manifesting with widespread chronic pain, disability, unemployment, and depression. He described his symptoms as burning pain in his hands, feet, elbows, and spine that began after a fall at work onto his upper back that left a rotator cuff tear and wrist injuries, along with chronic unexplained nausea severe enough to cause weight loss. His past medical history was notable only for unusual pain symptoms after accidental puncture of his left index finger with a wire brush, with disproportionate purple discoloration, coldness, and pain persisting for 10 years. His major medical history included learning disabilities, major depression, pulmonary micronodules, Peyronie’s disease, inguinal and abdominal hernias, and hypogonadism attributed to chronic opioid treatment for pain. There was no family history of neuropathy. He used prescribed gabapentin and oxycodone for pain management after morphine, topiramate, nortriptyline, and pregabalin were poorly tolerated.

**Figure 1.**
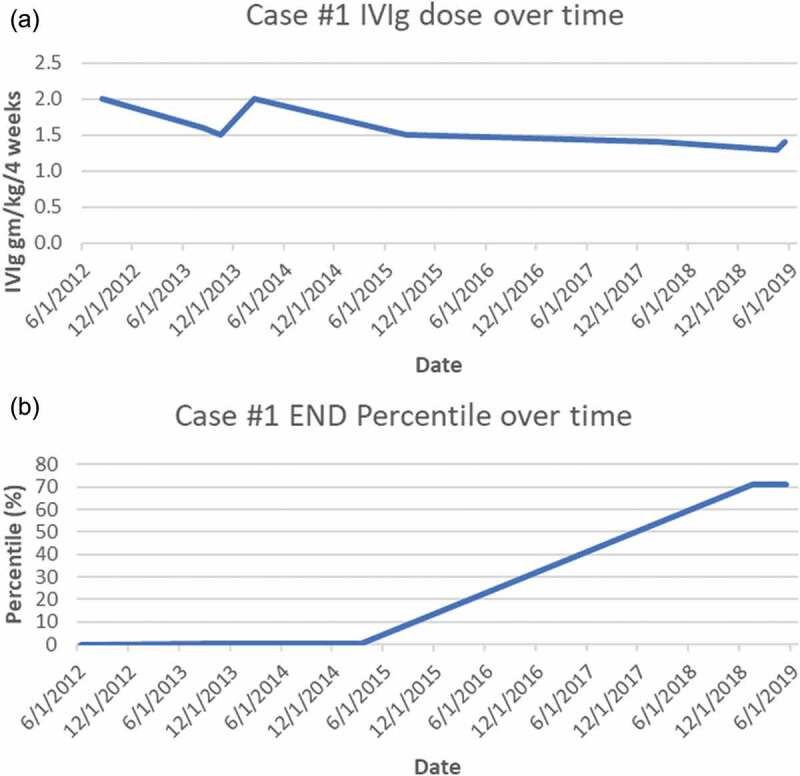
(a) Case 1 IVIg treatment doses (expressed as g/kg/4 weeks). (b) Case 1 END (% of predicted from sex-, race-, and age-adjusted normal distribution)

General and neurological exams revealed intact motor function and reflexes. His sensory exam was notable for distal reduction of pin-prick sensation below the mid-shins and wrists. Lumbosacral magnetic resonance imaging identified multilevel degenerative disease with mild canal stenosis and bilateral L4 root impingement. His lower leg skin biopsy revealed END of 45 neurites/mm^2^ skin surface area, below the first percentile of predicted and confirming a diagnosis of SFN (Figure 1). Comprehensive screening for causes of iiSFN revealed only repeated antinuclear antibodies (≥1:160 dilution) and repeatedly low complement C4 (11 mg/dL, 4 mg/dL, 8 mg/dL, 10 mg/dL).

Given his persisting severe confirmed SFN and inflammatory serologies, a trial of immunotherapy for likely autoimmune causality was discussed. Corticosteroids were judged as contraindicated by his mood disorder, so a 3-month trial of IVIg (2 g/kg/4 weeks, the typical initial dose for immune neuropathy^40^) was undertaken, and when IVIg reduced his pain and disability, treatment was continued. After 12 months of treatment, his sensory loss had improved, and his chronic nausea had resolved. Along with resuming exercise he regained 20 lb of lost weight. His repeat skin biopsy showed marginally improved END to 80 neurites/mm^2^, still less than first percentile of predicted. As his symptoms improved his IVIg was gradually tapered to 1.4 g/kg/4 weeks, but his clinical gains and END both regressed (to 59 neurites/mm^2^), so he requested resumption of 2 g/kg/4-week dosing, after which his symptoms again abated. He resisted further suggestions to taper until 36 months, when he agreed to and tolerated slower tapering. After 80 months of IVIg, his skin biopsy showed complete recovery of his END to the normal range (196 neurites/mm^2^, at the 71.4th percentile of predicted). We continued to search for other potential contributors to his SFN, and whole exome sequencing identified a heterozygous rare *SCN9A*-A3734G:p.Asp1245Ser variant exon 20, a cytoplasmic domain very near the N-terminus of the loop. The 1245A3734G SNP was a single nucleotide missense variant at a poorly conserved position in the protein, and the allele frequency was estimated at 0.0036 to 0.0055. Additionally, it has received conflicting interpretations of pathogenicity and has been reported in the literature in two individuals with erythromelalgia. Given this finding, mexiletine and oxcarbazepine were trialed but not tolerated.

## Case 2

A 32-year-old healthy male executive developed shingles on his right thorax. This resolved without complications after antiviral treatment, but it initiated disabling multisymptom illness interpreted as “chronic fatigue syndrome“ lasting 8 years at initial presentation to us. He had no significant prior medical history or family history of neuropathy. He had been physically fit and a regular runner before his zoster, but after his rash faded, he developed light-headedness during his runs, felt as if ”he had the flu,” and was unable to continue to exercise. His energy levels and cognitive abilities progressively decline until he became unable to work full time and at one point was barely able to leave his house. Other neuropathy symptoms included loss of sweating and hair growth from his lower legs and episodic paresthesias in his arms and legs that he did not consider painful. Additional dysautonomia symptoms included new postprandial bloating and mild constipation and new marked urinary urgency and frequency (12–20 times daily).

**Figure 2.**
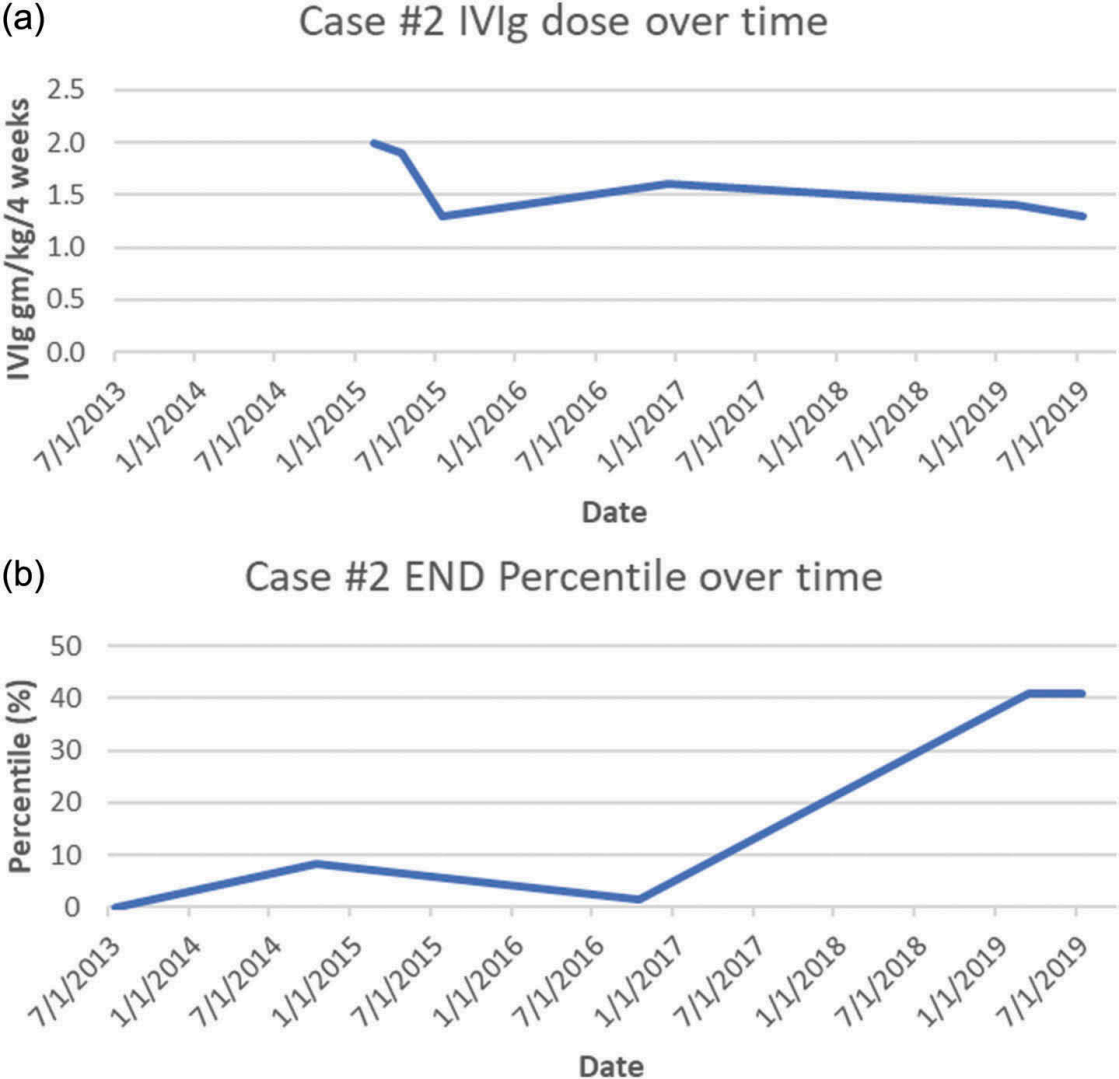
(a) Case 2 IVIg treatment doses (expressed as g/kg/4 weeks). (b) Case 2 END (% of predicted from sex-, race-, and age-adjusted normal distribution). *Note that the initial improvement in neurite density that occurred prior to initiation of IVIg corresponds to the initial trial of steroids that was cut short secondary to an adverse reaction

General and neurological examinations revealed only reduced pin-prick sensation below the mid-shins. A lower leg skin biopsy was very abnormal with only 82 neurites/mm^2^ (0.11th percentile of predicted), and a sural nerve biopsy performed for suspected inflammatory neuropathy revealed fascicles with minimal losses of normally myelinated large fibers and no excess cellularity. Electron microscopic examination of small fibers demonstrated widespread empty Schwann cell stacks, thus confirming a small fiber–predominant sensory neuropathy. Autonomic function testing identified POTS and reduced sweating at three sites, consistent with SFN. Invasive cardiopulmonary stress testing revealed low exercise duration of only 6.5 min. Oxygen uptake was abnormally low (62% of predicted with the anaerobic threshold only 32% of predicted VO_2_ max and the lactic acid threshold low at 17% of VO_2_ max). His maximum heart rate was 85% of predicted, although his biventricular ejection fraction increased normally with exercise. His pulmonary wedge pressure was 7 at rest and 22 at peak exercise. Ventriculographic cardiac output at rest was 8.65 L per minute, increasing to 18.5 L per minute. The study was interpreted as identifying that his exercise limits were not cardiac or pulmonary but most consistent with insufficient oxygen delivery, decreased oxygen extraction at peak exercise, and premature conversion to anaerobic metabolism. Comprehensive blood test screening for cause identified only antinuclear antibodies (1:160 titer) and autoantibodies against voltage-gated potassium channels that were interpreted as a nonspecific suggestion of disturbed immunity. Postinfectious autoimmunity causality was suspected, and because of prolonged serious disability, oral prednisone 1 mg/kg/day (70 mg/day) was initiated, with paresthesias, fatigue, and other symptoms rapidly improving and repeat biopsy improving to 143 neurites/mm^2^ (8.5th percentile, just above the 5th percentile  diagnostic cutoff). New increased intraocular pressure discovered during routine testing prompted early prednisone discontinuation and switch to IVIg initiated at 2 g/kg/4 weeks (140 g; Figure 2). His symptoms continued to improve steadily, with most ultimately resolving. He returned to full-time work, including a demanding international business travel schedule.

Nine months after starting IVIg, whole exome sequencing revealed two heterozygous *SCN9A* SNPs: A3310G:p.Ser1104Gly in exon 17, located centrally in a large cytoplasmic loop, slightly toward the N-terminal, plus a second in exon 18, T3414G:p.Asp1138Glu. This exon also encodes part of the central portion of the same large cytoplasmic loop, slightly toward the C-terminal. Because the A3310G SNP is in a region with highly conserved nucleotide and protein sequencing and very low prevalence (0.06%) in European and American populations, both sorting intolerant from tolerant (SIFT) computational analysis predicted it to be deleterious. The exon 18 variant had only been reported in a single individual at the time. Because the nucleotide was weakly conserved but the amino acid was a highly conserved amino acid, computational analyses gave conflicting predictions regarding pathogenicity. Neither had been reported in pain disorders.

IVIg taper after 15 months (to 1.3 g/kg/4 weeks) produced gradual return of symptoms, and a third skin biopsy performed 12 months into the taper revealed worse axonopathy (108 neurites/mm^2^, at the 1.64th percentile), so IVIg was increased to 1.6 g/kg/4 weeks. Improvement resumed and he began exercising again. After 4 years of immunotherapy his skin biopsy had entirely normalized to 175 neurites/mm^2^ (40.9 percentile of predicted), as had his previously abnormal autonomic function testing results. IVIg tapering was re-initiated more slowly with the intent of finding his minimally effective dose. His dose was initially tapered to 1.4 g/kg/4 weeks; 5 months later it was reduced to 1.3 g/kg/4 weeks, and 2 months later it was further reduced to 1.1 g/kg/4 weeks. After 2 months at the lowest dose, he felt that he had done better at a dose of 1.3 g/kg/4 weeks and was returned to that regimen.

## Discussion

To our knowledge, these are the first cases associating immunotherapy-responsive SFN with amino acid–substituting variant of unknown significance (VUS) in *SCN9A*, the chromosome 2q24 gene that encodes the α subunit of the Na_v_1.7 sodium channel implicated in familial and sporadic “idiopathic” SFN.^41^ Elegant in vitro electrophysiological studies in HEK293 cells and dorsal root ganglion cultures in the Yale laboratory of Dib-Hajj and Waxman showed that pathogenic *SCN9A* mutations cause hyperexcitability by either impairing slow inactivation, depolarizing slow and fast inactivation, or causing enhanced resurgent currents and increasing the number of action potentials evoked by depolarization. Persistently increased sodium channel activity can then trigger axonal degeneration, perhaps by reverse sodium–calcium exchange.^41^

These patients’ late onset and response of symptoms and pathology to IVIg are more consistent with inflammatory than genetic causality, but they do not preclude contributory or risk-modifying roles of their VUS. There is increasing recognition that classical fully penetrant Mendelian mutations are rare compared to more subtle deleterious consequences that can be conveyed by variants in critical genes such as *SCN9A*. Each person is estimated to carry 50 to 100 variants in genes previously implicated in genetic disorders.^42^ Predicting their functional consequences (rare variant analysis) requires increasingly complex algorithms. Relevant considerations include how stringently DNA and amino acid sequences are conserved in the population and the function of the affected DNA. Therefore, algorithms consider whether a variant changes an amino acid and, if so, how the altered protein differs in size, charge, and steric properties from the wild-type, with more differences indicating higher probability of pathogenicity. Rare variants that change highly conserved amino acids are more likely pathogenic, as are those that affect domains that bind externally or internally or regulate essential functions. The function of the affected protein is also important, and neuronal proteins that influence action potentials such as *SCN9A* have such critical roles that even minor alterations can be deleterious or fatal.^43^ Some genetic risk modifiers may only become relevant in individuals also exposed to other environmental or genetic risks or in specific ethnicities or locations.^44–46^ Recent mutations (sporadic cases with no family history) and those with incomplete penetrance are also underestimated.

Gene panels and whole exome sequencing can miss copy number variants and chromosomal rearrangements such as repeat expansions, insertions, deletions, and some translocations. Another limitation is that they miss effects caused by alterations in noncoding DNA sequences, including some that modulate DNA transcription and translation. Variants in non-protein-coding regions sometimes convey harm by altering biophysical properties of RNA genes or microRNAs to alter transcription or otherwise deter normal cell functioning.^45,47^ In addition, mitochondrial mutations, which are not captured, can be salient for small fiber and other polyneuropathies, including those associated with multiple lipomas.^48–52^ Whole genome sequencing will eventually replace gene panels and whole exome sequencing as prices continue to drop and evidence of its value increases.^53^

The current greatest limitation may be the lack of detailed phenotyping of the kind performed here. Insufficient or inaccurate phenotyping precludes confirming or refuting proposed potential genotype–phenotype associations. Large-scale consortia collection of genetic and standardized phenotypic data is required to aggregate enough reported affected and unaffected individuals with the same variants for computational analyses. The first was the Mendelian Inheritance in Man database first published as a book in 1966 by the Johns Hopkins pioneer Victor A. McKusick to catalog mendelian traits and disorders. In 1987 this migrated online as OMIM (Online Mendelian Inheritance in Man^54^) with the U.S. National Library of Medicine and was developed as a publicly accessible Internet resource by the U.S. National Center for Biotechnology Information in 1995 (https://www.ncbi.nlm.nih.gov/omim). The *SCN9A* entry is https://www.omim.org/entry/603415. Other databases compare a variant’s prevalence among patients with the expected phenotype with prevalence in the healthy population (risk association). The Broad Institute’s Exome Aggregation Consortium database analyzes variants from more than 60,000 genomes or exomes (*SCN9A*: http://exac.broadinstitute.org/gene/ENSG00000169432).^42^ In all, more than 30 predictive algorithms and databases (reviewed in de Beer et al.^55^) are available to predict pathogenic potential of minor allele variants, with most users combining results from several. A limitation is that the phenotypes are typically cross sectional, reported at one point in time, and thus do not adequately reflect the full natural history. Accuracy depends on numbers of phenotypes associated with a VUS and how penetrant it is. A variant may increase neuropathy risk 10-fold, say from 5% to 50%, but in that case, evaluating only three carriers of that VUS, even assuming 100% penetrance by age of assessment, still has a 6.25% risk of false-negative determination of nonpathogenicity. If the risk augmentation is less or age-of-onset older, the probability of false-negative predictions of pathogenicity increases further. Similarly, associating more than one case of an associated phenotype with VUS in a gene pathogenic for that phenotype cannot confirm causality unless the prevalence or the association is statistically higher than that in a large reference cohort of controls confirmed not to have the phenotype. Furthermore, it is not currently possible to predict whether two mutations that affect the same region of a protein (Case 2) could add to become pathogenic (compound heterozygosity). Given the complexity, the American College of Medical Genetics and Genomics and the Association for Molecular Pathology developed standards and guidelines for the interpretation of allele variants.^56^ Given the rapid pace of genetic discovery, we advise physicians and patients to request the actual “variant call files” DNA sequence files so that they can be re-compared to the genetic databases and publications every year or two.

The ClinVar public archive reports genotype/phenotype correlations and summarizes the frequencies of minor allele variants reported by various databases. The Inherited Neuropathy Consortium (INC) browser link for *SCN9A* is http://hihg.med.miami.edu/code/http/cmt/public_html/index.html#/ and the ClinVar listing is https://www.ncbi.nlm.nih.gov/clinvar/variation/331,972/#id_first. See Table 1 for the links to the SNPs posted in ClinVar and summaries of our analyses.

**Table 1.** Summary of VUSs

Case	Genomic variant	ClinVar link	Summary
1	NM_002977.3 (*SCN9A*): Exon 20:c.A3734G:p.Asp1245Ser	www.ncbi.nlm.nih.gov/clinvar/variation/130265/	Not reported in the INC browser and thus currently listed as of unknown significance for axonal hereditary sensory and autonomic neuropathy and “likely benign/uncertain significance” for the inherited erythromelalgia phenotype.
2 VUS 1	NM_002977.3 (*SCN9A*): Exon 17:c.A3310G:p.Ser1104Gly	https://www.ncbi.nlm.nih.gov/clinvar/variation/331,972/#id_first	Not reported in the INC browser and thus currently listed as “likely benign” for small fiber and axonal hereditary sensory and autonomic neuropathy and familial erythromelalgia because of no reported cases.
2 VUS 2	NM_002977.3 (*SCN9A*): Exon 18A:c.T3414G:p.Asp1138Glu	Not reported, link not available	Not reported to ClinVar or in the INC browser, but a survey of Na_v_ variants in painful and nonpainful neuropathies of all causes identified this in six patients and interpreted it as of uncertain significance.^36^

VUS = variant of unknown significance; INC = Inherited Neuropathy Consortium.

Potential interactions between genetics and inflammatory neuropathy, as we suggest here, are plausible although not yet well characterized. Non-Mendelian minor allele variants are increasingly shown to modify risk of developing neuropathy from other causes, including toxins, diabetes,^57^ injury, or separate deterministic mutations, with several linked to development of immune/inflammatory neuropathies.^58,59^ Variants in major histocompatibility complex alleles alter susceptibility to antibody-mediated disorders of the central nervous system and many other conditions, so involvement in immune peripheral nerve disorders is plausible.^60^ Minor variants in the *HLADq* genes that encode cell surface receptors are strong risks for autoimmune conditions, including celiac disease. They have also been associated with neuropathies including SFN.^61^ Indirect genetic risks have been identified as well; for instance, *MYD88* L265P is a common risk for Waldenström’s macroglobulinemia,^62^ which conveys risk for neuropathy, presumably from clonal autoantibodies targeting nerve epitopes.^63,64^ Mice deficient in the autoimmune regulator (*AIRE*) gene develop symtoms of Sjögren’s syndrome plus SFN detectable in the cornea and lachrymal glands.^65^ Given the strong risk that Sjögren’s poses for developing SFN,^21^ *AIRE* variants may prove to be human risk factors for SFN. Inflammatory mediators administered to cultures of dorsal root ganglia are newly reported to increase anterograde transport of vesicles containing Na_v_1.7 channels, delivering more to the distal axon, which raises channel density in the distal axolemma, presumably enhancing small fiber excitability.^66^

If these *SCN9A* VUSs did indeed contribute to these patients’ neuropathies, several potential explanations for their responses to immunotherapy should be considered. The possibility that patients’ improvement was a placebo effect was essentially eliminated given that all of their objective biomarkers for SFN normalized during their improvements in symptoms. Spontaneous unrelated contemporaneous remission coincidental with IVIg administration cannot be disproved, but it is extremely improbable after years without spontaneous improvement and the strong temporal associations between END values and immunotherapy dosing (see Figures 1 and 2). IVIg downregulates immune response not only by competitively inhibiting binding of circulating pathogenic autoantibodies but also by influencing T cells, cytokines, immune cell trafficking, complement, and Fc,receptors.^67^ IVIg has been proven effective by randomized controlled trials in other immune-mediated peripheral neuropathies,^68–70^ and it is increasingly used off-label for inflammatory central nervous system diseases including multiple sclerosis, autoimmune epilepsy, and paraneoplastic encephalitis. More research is needed to confirm the efficacy of IVIg in other neurological disorders, but its expense, scarcity, and the tremendous costs and logistic difficulties of conducting randomized trials (e.g., controlling for infusion reactions) remain substantial barriers.

We raise, but cannot answer, the question whether missense Na_v_ mutations might augment the probability of immune responses directed toward small fiber neurons, whether by changing surface antigens or through less direct mechanisms. All three VUSs reported here altered intracellular rather than extracellular proteins, so exposure of new epitopes seems unlikely, but other effects cannot be predicted. Theoretical models predict that many more risk variants remain unknown due to the current genome-wide significance threshold and that many different susceptibility loci, with very small effect sizes, can contribute to multifactorial disease. Polygenic risks are well described in multiple sclerosis,^71^ as is the wide phenotypic and genetic heterogeneity of Charcot-Marie-Tooth disease.^61^ Investigation of genetic influences on SFN is in its infancy, but large sets of real-world data are needed to advance research and inform treatment options. Therefore, the Massachusetts General Hospital Nerve Unit built a secure Internet interface for data sharing of rare genetic variants of known or potential significance for patients with SFN at https://neuropathycommons.org/content/neuropathy-gene-registry-0. With more VUSs analyzed, more will become “actionable,” meaning influencing diagnosis, prognosis, or treatment. Some may lead to gene-specific oligonucleotide or first precision therapies, as recently reported for hereditary sensory and autonomic neuropathy type 1.^62^ Even today, given the wide availability and low cost, combined with strong data for efficacy of sodium channel–blocking drugs, we consider it reasonable to consider empirically trying generally safe, potentially disease-modifying treatments such as the sodium channel blockers tried here in patients with potentially pathogenic VUS.
